# PET Imaging in Cardiac Sarcoidosis: A Narrative Review with Focus on Novel PET Tracers

**DOI:** 10.3390/ph14121286

**Published:** 2021-12-09

**Authors:** Petar Saric, Kathleen A. Young, Martin Rodriguez-Porcel, Panithaya Chareonthaitawee

**Affiliations:** Department of Cardiovascular Medicine, Mayo Clinic, 200 First Street SW, Rochester, MN 55905, USA; saric.petar@mayo.edu (P.S.); young.kathleen@mayo.edu (K.A.Y.); rodriguez.m@mayo.edu (M.R.-P.)

**Keywords:** cardiac sarcoidosis, positron emission tomography, fluorodeoxyglucose, somatostatin analogs, fluorothymidine, hypoxia tracers, methionine, pentixafor

## Abstract

Sarcoidosis is a multi-system inflammatory disease characterized by the development of inflammation and noncaseating granulomas that can involve nearly every organ system, with a predilection for the pulmonary system. Cardiac involvement of sarcoidosis (CS) occurs in up to 70% of cases, and accounts for a significant share of sarcoid-related mortality. The clinical presentation of CS can range from absence of symptoms to conduction abnormalities, heart failure, arrhythmias, valvular disease, and sudden cardiac death. Given the significant morbidity and mortality associated with CS, timely diagnosis is important. Traditional imaging modalities and histologic evaluation by endomyocardial biopsy often provide a low diagnostic yield. Cardiac positron emission tomography (PET) has emerged as a leading advanced imaging modality for the diagnosis and management of CS. This review article will summarize several aspects of the current use of PET in CS, including indications for use, patient preparation, image acquisition and interpretation, diagnostic and prognostic performance, and evaluation of treatment response. Additionally, this review will discuss novel PET radiotracers currently under study or of potential interest in CS.

## 1. Introduction

Sarcoidosis is a multi-system disease of unknown etiology characterized by the development of inflammation and noncaseating granulomas. Sarcoidosis has a predilection for the pulmonary system, but can involve nearly every organ system, including the heart [1]. The exact prevalence of sarcoidosis is unknown, with estimates ranging from 100 in 100,000 patients in a prospective cohort study of women aged 25 to 44 years, and up to 330 in 100,000 patients in some regions of the United States [2,3]. The rate of cardiac involvement by sarcoidosis (CS) is estimated to be between 20% to 70%, with CS accounting for approximately 25% of sarcoid-related mortality in the U.S. and up to 85% in the Japanese population [4,5,6,7,8]. CS can involve any part of the heart, most commonly involving the basal ventricular septum, left ventricular basal free wall, papillary muscles, and, to a lesser extent, the right ventricle [9,10]. Rarely, CS can present with pericardial and coronary artery involvement, presenting with pericardial effusion and myocardial ischemia and infarction, respectively [11,12,13]. Clinical presentation can range from no symptoms to conduction abnormalities, heart failure, arrythmias, valvular disease, and sudden cardiac death [7,14,15,16,17,18]. Additionally, up to 25% of patients with CS have isolated myocardial involvement, highlighting the fact that CS frequently exists in the absence of extracardiac disease [19,20]. Overall, the survival of patients with CS is variable, with 5-year survival ranging from 60% to 90% [21]. Previous studies have identified the presence of left ventricular dysfunction as the strongest independent predictor of mortality, with a 10-year survival of greater than 80% in those with a left ventricular ejection fraction ≥50%, compared to a 10-year survival of 19% in those with a left ventricular ejection fraction of <30% [17,22]. The leading cause of death among patients with CS is ventricular arrythmia, followed by congestive heart failure [15]. Given the high rate of cardiac involvement of sarcoidosis with the potential for poor outcomes, timely diagnosis of this condition is imperative. Unfortunately, traditional imaging modalities and histologic evaluation by endomyocardial biopsy often provide a low diagnostic yield, with the sensitivity of endomyocardial biopsy being around 20–30% due to the nonuniform myocardial distribution typical of CS [8,23]. Thus, there have been significant efforts to develop non-invasive imaging strategies for improved diagnosis of CS, as well as to provide prognostic information and assessment of therapeutic response. Cardiac positron emission tomography (PET) has emerged as a leading imaging modality for the diagnosis and management of CS. The purpose of this narrative review is to summarize several aspects of the current use of PET in CS, including indications for use, patient preparation, image acquisition and interpretation, diagnostic and prognostic performance, and evaluation of treatment response. Additionally, there will be a special focus on novel PET radiotracers currently under study or of potential interest in CS. PubMed was utilized to search for the articles and evidence cited in this manuscript, including those related to all aspects of the use of PET/CT for CS, treatment of CS, as well as new PET pharmaceuticals for imaging of CS.

## 2. Indications for Cardiac PET/CT for CS

^18^F-fluorodeoxyglucose (^18^F-FDG) is a glucose analog which is readily taken up by macrophages in areas of active inflammation, a process that is the pathophysiologic basis for ^18^F-FDG PET imaging in CS. ^18^F-FDG PET has been shown to be highly sensitive for the diagnosis of CS, with one systematic review and meta-analysis showing a sensitivity and specificity of PET for the diagnosis of CS of 89% and 78%, respectively [24,25,26,27]. The most frequently used guidelines for the diagnosis of CS are those from the Japanese Ministry of Health and Welfare (JMHW), which now include the use of cardiac PET in the diagnostic criteria following their update in 2017 [28,29]. The Heart Rhythm Society (HRS) 2014 guidelines for CS emphasize the importance of screening all patients with biopsy-proven extracardiac sarcoidosis with a careful history, an electrocardiogram, and echocardiography and have also included the use of cardiac PET as a diagnostic criterion in its diagnostic pathway [30]. Further efforts have been made to standardize the use of cardiac PET in the diagnosis and therapeutic monitoring of CS, with a 2017 joint statement from the Society of Nuclear Medicine and Molecular Imaging (SNMMI) and the American Society of Nuclear Cardiology (ASNC) outlining four scenarios in which cardiac PET/CT should be considered: (1) the presence of histologic evidence of systemic CS and one or more abnormal screening results for CS (electrocardiographic findings of complete left or right bundle branch block, unexplained pathologic Q waves in two or more leads, regional wall motion abnormalities and/or wall aneurysms on echocardiogram, thinning of the basal septum, left ventricular ejection fraction <50%, sustained or non-sustained ventricular tachycardia, cardiac magnetic resonance imaging (MRI) findings consistent with CS, and unexplained palpitations and/or syncope), (2) development of new-onset second- or third-degree heart block in a patient less than 60 years of age, (3) sustained idiopathic ventricular tachycardia, and (4) the need for serial PET/CT imaging in patients with diagnosed CS to assess response to treatment [31].

## 3. Patient Preparation for Cardiac PET/CT for CS

Myocardial metabolism is a dynamic process that can involve a mixture of energy sources, including glucose, ketones, amino acids, and free fatty acids [32]. Under normal physiologic conditions, there is often physiologic myocardial glucose utilization, thus the potential for ^18^F-FDG uptake that is not due to cardiac inflammation. In order to unmask ^18^F-FDG uptake that is only due to inflammation from CS and thus improve the specificity of ^18^F-FDG PET imaging for the diagnosis of CS, it is imperative to achieve adequate suppression of background physiologic ^18^F-FDG uptake by cardiac myocytes. Several approaches have been developed to suppress physiologic ^18^F-FDG uptake, including prolonged fasting, dietary manipulation, and administration of unfractionated heparin.

In the fasting state, lipids are utilized as the preferred cardiac myocyte energy substrate instead of glucose [33]. Previous studies have shown success rates of 62% to 90% with fasting protocols for suppression of physiologic myocardial ^18^F-FDG uptake [24,34,35,36]. There are several limitations to this approach, including potential for poor adherence with prolonged fasting, as well as the risk of hypoglycemia. Additionally, Masuda and colleagues found that 38% of participants still showed evidence of physiologic ^18^F-FDG uptake despite undergoing an 18 h fast [35].

Alternatively, physiologic myocardial ^18^F-FDG uptake can be suppressed by implementing a high-fat, low-carbohydrate diet, which facilitates the transition from glucose to fatty acid metabolism in cardiac myocytes. One study has shown this approach to be superior to prolonged fasting alone, with 67% of patients in the diet group achieving adequately suppressed physiologic ^18^F-FDG uptake, compared with 52% in a group that underwent a 12 h fast [37]. Despite the potential for improved suppression of physiologic ^18^F-FDG uptake with this approach, nonspecific myocardial uptake may still be seen in up to 20% of patients [38].

Administration of unfractionated heparin has also been used to increase serum free fatty acid levels through induction of lipolysis, in an attempt to lower glucose use by the myocardium [39]. A commonly used protocol is the administration of 50 IU/kg of unfractionated heparin intravenously fifteen minutes before administration of ^18^F-FDG [34,38]. There have been conflicting data on the use of unfractionated heparin, however, and the utility of this approach is uncertain [34,35,40].

The SNMMI/ASNC 2017 joint expert consensus outlines several recommendations regarding patient preparation prior to cardiac PET/CT for cardiac sarcoidosis [31]. Regarding dietary modification and fasting, two options are recommended. The preferred recommendation is for the consumption of at least two high-fat (>35 g), low-carbohydrate (<3 g) meals the day prior to the study and then fasting for 4–12 h. In a recent study, Christopoulos and colleagues demonstrated that strict adherence to this protocol with nursing follow up for reinforcement of dietary instructions and dietary log review lead to the suppression of physiologic myocardial ^18^F-FDG uptake in 91% of patients, compared to 78% in patients when nursing follow up was not implemented [41]. An alternative regimen is for the patient to fast for >18 h prior to the study [31]. If unfractionated heparin is to be administered, the expert panel recommends the use of a single 50 IU/kg intravenous bolus of unfractionated heparin to be given fifteen minutes before ^18^F-FDG administration.

## 4. Acquisition of Cardiac PET/CT Images for CS

**Figure 1:**
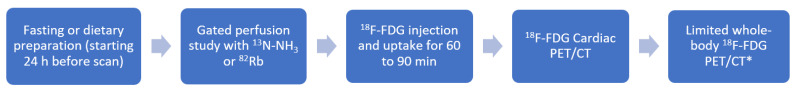
Outline of the typical PET protocol for evaluation of CS. * If there is clinical suspicion of extracardiac CS, include at least the chest, liver, and spleen.

The SNMMI/ASNC 2017 joint expert consensus statement recommends obtaining two sets of resting images to assess the spectrum of CS: a myocardial perfusion image (MPI) acquired using either ^13^N-NH_3_ or ^82^Rb, followed by a cardiac ^18^F-FDG image. Caution must be used to avoid overcalling lateral perfusion abnormalities with the use of ^13^N-NH_3_ on perfusion images, as this may be a normal finding with this radiotracer. If PET myocardial perfusion imaging is not available, SPECT MPI with ^201^Tl or ^99m^Tc-labeled radiotracers may be used as a substitute. If using SPECT MPI in place of PET MPI, the use of attenuation correction is recommended to reduce artifact. Acquisition of gated perfusion images is crucial for assessment of regional wall motion abnormalities and left ventricular systolic function, which provides important diagnostic and prognostic data. Following the perfusion study, ^18^F-FDG is injected, with an uptake period of 60 to 90 min, after which nongated dedicated cardiac emission images are acquired over 10 to 30 min, depending on scanner specifications, administered radiotracer dose, and patient characteristics [42]. The typical PET protocol for CS is outline in Figure 1.

Consideration should be given to extending the field of view for the ^18^F-FDG PET study to include at least the chest, liver and spleen, if there is clinical suspicion for extracardiac sarcoidosis, or if ^18^F-FDG PET evaluation for extracardiac disease has not been recently performed. Identification of extracardiac involvement may add important diagnostic, prognostic, and therapeutic implications, and may also help to identify potential targets for biopsy if needed.

## 5. Interpretation of Cardiac PET/CT Images for CS

Prior to the interpretation of the cardiac PET/CT study for CS, a thorough examination of the patient’s medical record should be performed, including review of pertinent history, as well as previous diagnostic studies and therapeutic interventions with an emphasis on identifying non-sarcoid causes of myocardial perfusion and FDG abnormalities that may confound image interpretation. Additionally, patient adherence to adequate dietary preparation prior to the PET study should be confirmed. Interpretation of images begins with the assessment of quality-control, including confirmation of proper coregistration between CT and PET images, as misalignment can occur for a variety of reasons [42,43]. In a previous study, up to 40% of cardiac PET/CT scans were found to show false-positive perfusion abnormalities due to misregistration, with over half of the false-positive defects quantified as moderate to severe, highlighting the importance of proper alignment and coregistration of images [44]. Additional quality-control steps include confirmation of adequate suppression of myocardial physiologic ^18^F-FDG uptake, defined as no visible ^18^F-FDG uptake or uptake that is lower than that of the blood pool [38].

**Figure 2:**
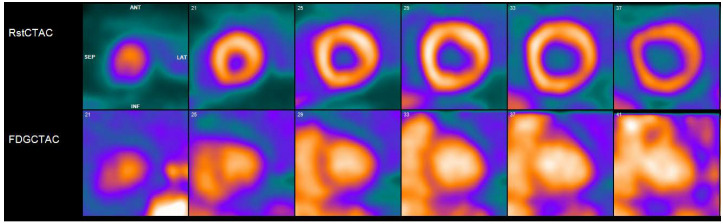
Cardiac positron emission tomography/computed tomography (PET/CT) with ^13^N-ammonia (top row) and ^18^F-flurodeoxyglycose (FDG) (bottom row) demonstrates normal resting perfusion and adequate myocardial suppression with no evidence of FDG uptake to suggest active inflammatory state.

**Figure 3:**
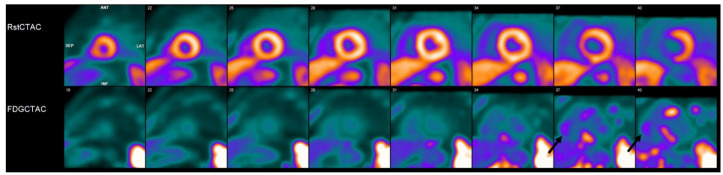
Cardiac positron emission tomography/computed tomography (PET/CT) with ^13^N-ammonia (top row) and ^18^F-flurodeoxyglycose (FDG) (bottom row) demonstrates a perfusion defect involving the basal septum with corresponding abnormal FDG uptake consistent with an inflammatory process. This patient had biopsy-proven systemic sarcoidosis, with history of complete heart block requiring pacemaker implantation. Findings were therefore felt to be consistent with probable cardiac sarcoidosis. In addition, patient was noted to have abnormal FDG uptake in the right ventricular free wall (black arrows), which portends a worse prognosis.

After appropriate quality-control assessment has been performed, the PET perfusion and ^18^F-FDG images should be interpreted simultaneously in the standard cardiac planes (short axis, vertical long axis, and horizontal long axis). A normal cardiac PET study for CS will show normal resting myocardial perfusion in addition to absence of myocardial ^18^F-FDG uptake, indicating the absence of active inflammation and complete suppression of physiologic ^18^F-FDG myocardial uptake (Figure 2). Inadequate suppression of physiologic ^18^F-FDG uptake, which is often due to suboptimal patient preparation, could show a pattern of diffuse homogenous ^18^F-FDG myocardial uptake. A pattern of focal areas of ^18^F-FDG uptake, with or without anatomically corresponding perfusion defects, is suggestive of active inflammation in the appropriate clinical setting (Figure 3). The presence of a perfusion defect, in the absence of another cause for decreased perfusion such as coronary artery disease, without any associated ^18^F-FDG uptake, is consistent with scarring. The presence of myocardial ^18^F-FDG uptake on the normalized cardiac display should be confirmed on the hybrid general nuclear medicine display, because the normalized cardiac display can lead to artifactual accentuation of areas of mild ^18^F-FDG uptake when viewed in a normalized fashion.

It is important to note that focal uptake in the lateral wall without an associated perfusion defect may be a nonspecific finding. Additionally, in patients with pacemaker or implantable cardiac defibrillator leads, images should be reviewed with and without attenuation correction, as ^18^F-FDG around device leads on attenuation-corrected images may be due to over-correction associated with metallic implants due to the energy-dependent extrapolation of the attenuation coefficients [45]. Lastly, even with rigorous metabolic preparation and heparin use, nonspecific myocardial ^18^F-FDG uptake may still occur, highlighting the importance of the comprehensive evaluation of all available data including consideration of additional advanced imaging modalities, such as cardiac MRI.

In addition to qualitative interpretation of the cardiac PET study for CS, semi-quantitative assessment using ^18^F-FDG standard uptake value (SUV) metrics can be helpful in evaluating the severity of pretreatment inflammation, as well as for responses to therapy [31]. Quantification of myocardial inflammation by SUV and other quantitative metrics is an area under active investigation, with no currently identified SUV cutoff that can delineate normal myocardium from CS and no data to support the use of one method over another [46].

## 6. Diagnostic and Prognostic Performance of Cardiac PET/CT for CS

Several recent meta-analyses have evaluated the diagnostic accuracy of ^18^F-FDG PET/CT for CS. In 2012, Youssef and colleagues performed a meta-analysis of 7 studies that included a total of 164 patients, with ^18^F-FDG PET/CT showing a sensitivity and specificity of 89% and 78%, respectively, for CS [27]. More recently, Kim and colleagues analyzed 17 studies involving 891 patients, reporting a pooled sensitivity of 84% and a specificity of 83%; there was considerable heterogeneity of sensitivity and specificity results between the included studies, mainly due to methodological differences [47]. There have been several studies evaluating the prognostic value of ^18^F-FDG PET/CT for CS. In 2014, Blankstein and colleagues evaluated a cohort of 118 patients with suspected or known CS who underwent ^18^F-FDG PET/CT imaging, finding an almost fourfold increase in death and malignant arrythmias at 1.5 year follow up in patients who had abnormalities on both perfusion and ^18^F-FDG PET images [48]. Additionally, right ventricular ^18^F-FDG uptake was also associated with a significant increase in mortality and ventricular arrythmias, with a hazard ratio of 4.22, although right ventricular ^18^F-FDG uptake was rare [48]. In a study of 38 patients who underwent ^18^F-FDG PET/CT for CS, Ahmadian and colleagues found cardiac metabolic activity, measured by SUV, to be a predictor of adverse events, providing further evidence for the prognostic value for ^18^F-FDG PET/CT in the evaluation of CS [49]. Several additional studies have added to the evidence supporting the prognostic value of SUV [46,50,51]. Additional quantitative variables, such as summed rest scores in segments showing a perfusion-metabolism mismatch and the coefficient of variation of ^18^F-FDG uptake have also been shown to provide prognostic information in patients undergoing ^18^F-FDG PET for CS [52]. More recently, Subramanian and colleagues demonstrated that a novel SUV-based metric, the ^18^F-FDG uptake index (UI), defined as the product of maximum left ventricular SUV and the number of left ventricular segments with abnormal uptake, was an independent predictor of treatment response [53].

## 7. Cardiac PET/CT for Assessment of Treatment Response in CS

The potential of serial ^18^F-FDG PET/CT studies to provide information on treatment response in CS is of significant value and current interest. In a study of 23 patients undergoing treatment with CS, Osborne and colleagues found that a reduction of myocardial inflammation, as measured by SUV, on serial ^18^F-FDG PET/CT scans was associated with an improvement in left ventricular ejection fraction [54]. This finding was also seen by Muser and colleagues, in a study of 20 patients with CS and ventricular tachycardia who underwent catheter ablation [50]. In addition, they found that lack of reduction in myocardial inflammation by SUV on serial imaging studies was associated with an almost twentyfold (hazard ratio 18.96) increase in risk of major adverse cardiac events, including death, cardiac transplantation, heart failure hospitalization, and implantable cardioverter-defibrillator therapies [50].

## 8. Treatment of CS

Treatment of patients with CS is challenging. Immunosuppressive therapy for CS is not without risk and thus it is imperative to use all available data, including information from alternative imaging modalities, such as cardiac MRI when available, when making the diagnosis of CS. As discussed previously, adequate suppression of physiologic ^18^F-FDG by the myocardium is imperative before cardiac PET/CT to minimize false-positive results which may lead to unnecessary treatment. A multidisciplinary approach including cardiology, electrophysiology, rheumatology, and often pulmonology is necessary in the care of this challenging patient population.

Currently, corticosteroids are the mainstay of therapy for CS, although there are limited data on the ideal drugs, doses, or duration of treatment, and there is variance in agreement on who should and should not be treated. In general, treatment is recommended in patients with symptomatic disease and evidence of active inflammation, whereas treatment in asymptomatic patients with evidence of active inflammation is more controversial. Some experts recommend treatment of asymptomatic individuals with evidence of active cardiac inflammation to prevent myocardial injury and disease progression, but there is no consensus on treatment or definitive data in these patients. Additionally, there is limited data on outcomes with corticosteroid treatment. One small study of 20 patients with CS and atrioventricular block showed a significant decline in left ventricular ejection fraction and increased incidence of ventricular tachycardia in patients who did not receive treatment with corticosteroids compared to those who did over a mean follow up period of approximately 80 months [55]. In a retrospective study of 43 patients with CS, Chiu and colleagues found that long-term treatment with prednisolone showed preventative effects for left ventricular remodeling and ejection fraction when started early in the course of the disease [22]. The data for use of alternative immunosuppressive agents is quite limited. Ballul and colleagues, in a study of 36 patients with symptomatic CS, observed that patients treated with steroids plus an immunosuppressant (either azathioprine, methotrexate, or cyclophosphamide) had significantly lower rates of relapse compared to those who received steroids alone (16.7% versus 45.8%) over a median follow up interval of 3.6 years [56]. In another recent study of 34 patients, the use of serial cardiac PET was shown to be beneficial in guiding dose adjustments in those receiving chronic steroid therapy for CS, with almost half of included patients able to be weaned off steroids after 1 year while maintaining good disease control [57]. More recently, several biologic agents have been evaluated in the treatment of CS. Infliximab, a chimeric monoclonal antibody that inhibits tumor necrosis factor alpha, has emerged as a potential therapy for CS, with a recent study of 22 patients with refractory CS on standard therapy showing a significant reduction in cardiac inflammation on cardiac PET and improvement in left ventricular function after addition of infliximab [58]. Another agent with potential utility in CS is rituximab, a chimeric monoclonal antibody targeting CD20 on B cells. Recently, Elwazir and colleagues retrospectively evaluated seven patients with treatment refractory CS who received therapy with rituximab, finding that six out of seven patients receiving rituximab showed significantly decreased inflammation on ^18^F-FDG PET/CT imaging by SUV [59]. Many unanswered questions remain regarding the optimal treatment of CS, and there are currently several ongoing trials which hope to add data to the field. The CHASM-CS study (NCT03593759), currently in the patient recruitment phase is the first randomized controlled trial in CS. This trial will aim to evaluate treatment with prednisone alone versus a prednisone taper and methotrexate therapy for 6 months, on several outcomes in patients with symptomatic CS. Another randomized controlled trial, also in the recruitment phase, is MAGiC-ART (NCT04017936). This trial will aim to evaluate anakinra, an interleukin-1 antagonist, in the treatment of CS.

## 9. New PET Radiopharmaceuticals for Imaging of Inflammatory Diseases

The use of ^18^F-FDG PET/CT in the imaging of inflammation in CS has seen significant growth. However, despite various approaches aimed at the suppression of physiologic myocardial ^18^F-FDG uptake, up to 10% to 15% of patients may have an inconclusive study [38]. If inadequate suppression is not recognized, patients may receive unnecessary treatment and thus be exposed to potentially significant medication side effects. If inadequate myocardial ^18^F-FDG suppression is recognized, this may lead to repeat imaging, leading to physician and patient dissatisfaction, adverse financial consequences, and additional patient radiation exposure [30,35,39]. Thus, there is a need for the development of new radiopharmaceutical tracers that do not require dietary preparation and that lack physiologic uptake by cardiac myocytes. In recent years, there have been significant advances in novel molecular imaging techniques for inflammatory disorders, with potential applications in many conditions, including CS. The development of several new radiopharmaceutical agents with the potential for more specificity than ^18^F-FDG, a marker of gross inflammation, has created tremendous opportunity for advances in the diagnosis and treatment of CS and other cardiomyopathies.

Current molecular imaging approaches with potential usefulness in the evaluation of infiltrative and inflammatory cardiomyopathies target several aspects of the pathophysiologic processes in these diseases. One approach is the targeting of the specific infiltrate itself, such as inflammatory noncaseating granulomas in CS. Molecular imaging can also be used in assessment of the indirect effects of these disease processes, targeting their functional, metabolic, and physiological consequences [60]. As many of these new radiopharmaceutical agents move closer to clinical use, there will be a need to familiarize clinicians with their potential applications in patient care. The remainder of this review will focus on highlighting several novel radiopharmaceutical agents and imaging targets. Table 1 summarizes the novel PET radiotracers that will be discussed in this article.

## 10. Radiolabeled Somatostatin Analogs

**Figure 4:**
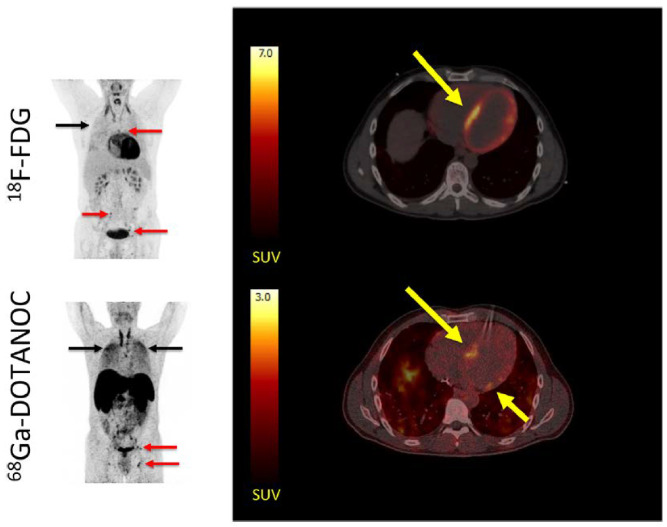
MIPs (left) showing multiple ^18^F-FDG and ^68^Ga-DOTANOC avid lymph nodes (red arrows) and lung tissue (black arrows) in a patient with both cardiac and systemic sarcoidosis. Transaxial slices of the heart (right) showed a focal on diffuse pattern of ^18^F-FDG uptake (top right), which was rated as inconclusive by all reviewers, compared to all reviewers rating the ^68^Ga-DOTANOC uptake (bottom right) as pathologic in the septum. Reprinted from ref. [65].

An alternative target in PET/CT for CS that is currently of great interest is the somatostatin receptor (SSTR). Five subtypes of the SSTR, all G protein-coupled receptors, have been described, with the SSTR 2 subtype showing potential promise in PET/CT assessment of CS. SSTR 2 has been shown to be the most frequently expressed SSTR subtype on activated lymphocytes, and has been found to be overexpressed in sarcoid granulomas [61]. Early studies evaluating the feasibility of SSTR-targeted imaging in sarcoidosis were done using ^111^In-pentetreotide scintigraphy, which showed better performance compared to gold-standard imaging with ^67^Ga-scintigraphy, as well as potential for disease activity monitoring [62,63]. More recently, interest has shifted to PET SSTR-targeted tracers, with ^68^Ga-DOTATATE, ^68^Ga-DOTANOC, and ^68^Ga-DOTATOC being the most commonly used analogues [64]. ^68^Ga-somastatin analogues target multinucleated cells and activated macrophages that express SSRT 2, which is not present on normal cardiac myocytes [65]. The lack of physiologic myocardial uptake of ^68^Ga-somastatin analogues potentially allows for a high signal-to-background ratio for PET/CT imaging, which may improve diagnostic specificity (Figure 4). Lapa and colleagues conducted a feasibility study involving 15 patients with biopsy-proven sarcoidosis with suspicion for CS, finding the overall concordance between cardiac MRI and ^68^Ga-DOTATOC PET/CT to be 96.1% for detection of abnormalities based on the American Heart Association 17-segment model of the left ventricle [66]. In a dual tracer study of ^18^F-FDG PET/CT and ^68^Ga-DOTANOC PET/CT in 19 patients with suspected CS, Gormsen and colleagues found the diagnostic accuracy of ^68^Ga-DOTANOC PET/CT to be 100%, compared to 79% for ^18^F-FDG PET/CT [65]. Additionally, there was less interobserver variability in the ^68^Ga-DOTANOC PET/CT group [65].

Several obstacles remain to wider adoption of SSTR-targeted PET imaging for CS. Results from a small study of 13 patients with suspected CS based on positive ^18^F-FDG PET/CT results found concordance of ^18^F-FDG and ^68^Ga-DOTATATE myocardial uptake to only be 54% [67]. In that study, three explanted hearts were examined by immunohistochemistry for expression of SSTR 2 positivity in sarcoid granulomas, showing only weak staining, with no significant staining of normal myocardium [67]. Currently, SSTR-targeted imaging appears to be the most promising alternative imaging modality under investigation for CS; however, significant challenges remain, and more research in this area is needed.

## 11. Proliferation Tracers: Radiolabeled Thymidine and Choline Analogs

**Figure 5:**
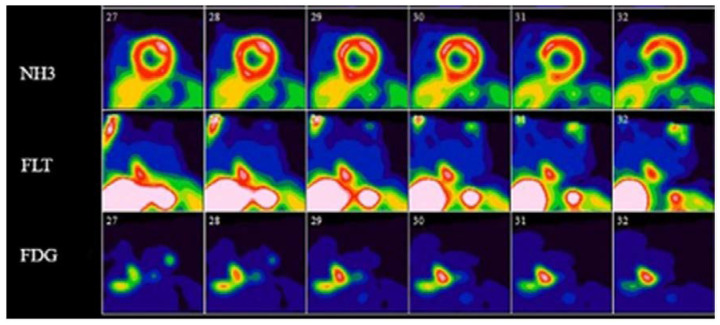
Short axis ^13^N-ammonia perfusion, ^18^F-FDG-, and ^18^F-FLT-PET images in a patient with active cardiac sarcoidosis. ^13^N-ammonia perfusion images (top row) show scarring of the inferior ventricular septum, with both FDG- and FLT-PET images showing evidence of active cardiac sarcoidosis in this area. Reprinted from ref. [75].

3′-deoxy-3′-^18^F-fluorothymidine (^18^F-FLT), a radiolabeled thymidine analog, initially developed as a PET tracer for use in the imaging of cellular proliferation in vivo, is another radiotracer with promising potential in PET for CS [68]. ^18^F-FLT is trapped intracellularly by the activity of thymidine kinase 1, an enzyme that is 10-fold more active during cellular proliferation, and has previously been shown to accumulate in granulomas with proliferative inflammation [69,70,71]. ^18^F-FLT lacks physiologic uptake by cardiac myocytes and does not require the extensive dietary preparation that is necessary with ^18^F-FDG PET/CT imaging [72,73]. In a study of 20 patients with sarcoidosis with and without cardiac involvement, Norikane and colleagues demonstrated that ^18^F-FLT PET/CT can detect cardiac sarcoidosis as well as ^18^F-FDG PET/CT, with a sensitivity of 85%, specificity of 100%, and accuracy of 90% for ^18^F-FDG PET/CT, compared with a sensitivity of 92%, specificity of 100%, and accuracy of 95% for ^18^F-FLT PET/CT [73]. Additionally, no ^18^F-FLT PET/CT studies were read as inconclusive, compared with 20% of ^18^F-FDG PET/CT scans that were rated as inconclusive [73]. Mean SUV_max_, however, was significantly lower with ^18^F-FLT when compared to ^18^F-FDG, perhaps reflecting the propensity of ^18^F-FLT to identify areas of cellular proliferation, compared to both cellular proliferation and inflammation with ^18^F-FDG [73]. Another small study of 14 patients by Martineau and colleagues showed excellent agreement between ^18^F-FLT PET/CT and ^18^F-FDG PET/CT for diagnosis of CS, with excellent interobserver agreement with ^18^F-FLT that was comparable to that of ^18^F-FDG [74]. Figure 5 highlights an example of a ^13^N-ammonia perfusion PET, ^18^F-FDG-, and ^18^F-FLT-PET study in a patient with active CS [74]. In another study, this same group showed ^18^F-FLT PET SUV_total_ to correlate strongly with sum rest scores by PET perfusion imaging done with ^13^N-ammonia or ^82^Rb, indicating that ^18^F-FLT PET/CT may provide different information than ^18^F-FDG PET/CT, such as the presence of myocardial scarring [75]. These small studies have demonstrated that ^18^F-FLT PET/CT may be a useful alternative to ^18^F-FDG PET/CT, although further data will be necessary before widespread use is recommended.

Another group of proliferation tracers with potential for future application in CS, most often used in oncology, are choline analogs, which include ^11^C-choline, ^18^F-choline, and ^18^F-fluoroethyl-choline [72]. Radiolabeled choline analogs are taken up in the synthesis of phosphatidylcholine, a cell membrane component, and thus can potentially serve as a marker of cellular proliferation. There are several reports of incidental uptake of these tracers in CS as well as systemic sarcoidosis in PET imaging done for other indications [76,77,78]. Choline analogs are not known to be taken up physiologically by normal cardiac myocytes.

Lastly, there are a few recent case reports documenting the potential of 4′-[methyl-^11^C]-thiothymidine (4DST), another thymidine analog, for application in CS [79,80]. Minamimoto and colleagues recently published several cases demonstrating the excellent performance of 4DST PET compared to ^18^F-FDG PET in the identification of active CS lesions, as well as in the assessment of treatment response [79]. Additional advantages of this tracer included short scan duration, with a 10-min PET scan being performed 20 min after 4DST administration, as well as low physiologic myocyte uptake and no need for dietary preparation.

## 12. Hypoxia Tracers: Radiolabeled Nitroimidazoles

**Figure 6:**
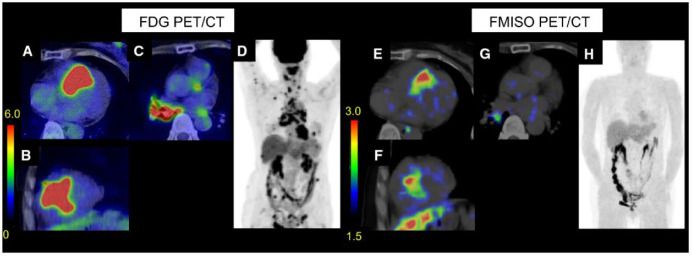
Example of ^18^F-FDG PET/CT and ^18^F-FMISO PET/CT images in a patient with cardiac sarcoidosis. Transverse, short-axis, and maximum intensity projection (MIP) images are provided. Significant abnormal cardiac ^18^F-FDG uptake was seen, as well as abnormal uptake in lymph nodes in the neck, mediastinum, hilum, abdomen, and inguinal lymph nodes (**A**–**D**). ^18^F-FMISO showed corresponding abnormal cardiac uptake, as well as abnormal uptake in a right hilar lymph node (**E**–**H**). Accessed on 11 July 2021. Modified and reprinted with permission from ref. [86]. Copyright 2021 Furuya, S. et al.

Radiolabeled nitroimidazoles, initially developed as tissue hypoxia imaging probes with applications in oncology, are of growing interest as potential alternative radiotracers in the imaging of CS [81]. ^18^F-fluoromisonidazole (FMISO), the first developed radiolabeled nitroimidazole for use in PET imaging, has been shown to selectively accumulate in hypoxic cardiac myocytes [82]. Nitroimidazoles enter cells through passive diffusion and are reduced in hypoxic cellular environments into reactive species, becoming trapped in hypoxic cells [83,84]. ^18^F-FMISO localizes to areas with upregulation of hypoxia-inducible factor, which has been shown to be overexpressed in sarcoid granulomas [85]. The current data for use of ^18^F-FMISO in CS is limited. A recent study by Furuya and colleagues found concordant results by ^18^F-FDG PET/CT and ^18^F-FMISO PET/CT in 8 out of 9 patients with CS, with no physiologic ^18^F-FMISO uptake noted [86]. Additionally, the ^18^F-FDG SUV_max_ values and metabolic volumes of ^18^F-FMISO-positive lesions were significantly higher when compared to ^18^F-FMISO-negative lesions [86]. Figure 6 provides an example of ^18^F-FDG PET/CT and ^18^F-FMISO PET/CT images in a patient with CS [86]. There have been several limitations to the cardiac applications of ^18^F-FMISO PET imaging, including slow clearance, limited first-pass uptake, and high liver uptake, which results in low target-to-background image contrast and requires administration of the tracer several hours prior to the imaging study [87]. Several next-generation nitroimidazole PET radiotracers are currently under development, with the goal of improving blood clearance and image contrast [88,89].

## 13. Radiolabeled Amino Acid Compounds: ^11^C-Methionine

^11^C-methionine, used in the molecular imaging of amino acid metabolism, is a PET tracer with some limited data in multitracer studies combined with ^18^F-FDG that has shown usefulness in the differentiation of malignant from benign tumors, particularly granulomas [71]. This is due to lower uptake of ^11^C-methionine compared to ^18^F-FDG by inflammatory lesions such as granulomas, compared to similar uptake of both agents by malignant tumors [71,90]. The use of ^11^C-methionine in humans for the evaluation of sarcoidosis is currently limited to a few case reports in which uptake was seen on ^11^C-methionine PET scans in patients with pulmonary and neurosarcoidosis [91,92]. Additionally, in a study evaluating an experimental animal model of autoimmune myocarditis, ^11^C-methionine demonstrated colocalization with ^18^F-FDG uptake in proven inflammatory lesions, while also showing a lack of physiologic uptake in normal myocardium [93]. To date, there are no studies evaluating the use of ^11^C-methionine in CS.

## 14. Radiolabeled CXCR4 Receptor Ligand

C-X-C motif chemokine receptor 4 (CXCR4), a leukocyte G-protein-coupled receptor that is involved in several inflammatory and autoimmune disorders, has also been shown to play a role in the mediation and resolution of inflammation in patients with acute myocardial infarction (MI) [94,95]. ^68^Ga-pentixafor is a radiolabeled CXCR4 ligand initially used in oncologic PET imaging [96,97]. Several recent studies have demonstrated the usefulness of ^68^Ga-pentixafor in PET imaging of atherosclerosis and post-MI inflammation [98,99,100]. To our knowledge, PET imaging of CXCR4 receptors with radiolabeled ligands, such as ^68^Ga-pentixafor, has not been investigated for potential application in CS. However, due to the specificity of ^68^Ga-pentixafor for the CXCR4 receptor and lack of significant physiologic myocardial uptake, this pathway is an intriguing area for potential future investigation.

## 15. Summary

Despite advances in non-invasive imaging modalities, the diagnosis of CS remains challenging. Prompt diagnosis of CS is imperative due to the significant morbidity and mortality associated with this disease, as well as the implications for therapy, monitoring, and prognosis. ^18^F-FDG PET/CT has emerged as the leading imaging modality for use in CS, although there remain several disadvantages to its use, including issues related to physiologic myocardial ^18^F-FDG uptake which can lead to inconclusive studies. Despite the use of dietary preparation protocols to minimize these issues, there remains a need for the development of new radiopharmaceutical PET tracers that lack physiologic myocardial uptake and thus do not require extensive patient preparation. In recent years, several new molecular imaging targets have been proposed as alternatives to ^18^F-FDG, with SSTR-targeted PET analogs having the most, although still limited, data at this time. Current data for the non-FDG PET radiotracers discussed is limited to small studies, and larger prospective studies will be needed in the future to further ascertain their potential role in the diagnosis and management of CS.

There are several important areas where future research is needed in CS. Validation of methods for quantification of cardiac inflammation continues to evolve, with growing data supporting the use of SUV metrics. Moving forward, the role of SUV metrics for quantification of cardiac inflammation will likely continue to expand. Additionally, several other areas of ongoing investigation may hold future promise in CS. Alternative imaging techniques, such as hybrid cardiac PET/MRI, may provide further information on prognosis and therapeutic response, as well as improved diagnostic accuracy [101]. Quantification of myocardial blood flow and myocardial flow reserve by PET imaging, is another promising technique under investigation, with a previous small study showing reductions in these parameters in patients with active CS [102]. Lastly, the optimal timing of follow-up imaging beyond the initial diagnosis of CS is unknown and further research is needed for further clarification.

## Figures and Tables

**Table 1:** Novel Radiopharmaceutical PET Agents for Imaging in Cardiac Sarcoidosis.

Proposed Agent	Example(s)	Mechanism	Production	Half-Life
Radiolabeled somatostatin analog	^68^Ga-DOTATATE; ^68^Ga-DOTANOC; ^68^Ga-DOTATOC	Targets multinucleated cells and activated macrophages that express somatostatin receptor (SSTR) 2, which is not present on normal cardiac myocytes.	Generator	68 min
Radiolabeled thymidine analogs	3′-deoxy-3′-^18^F-fluorothymidine (^18^F-FLT); 4′-[methyl-^11^C]-thiothymidine (4DST)	^18^F-FLT is trapped intracellularly by the activity of thymidine kinase 1, which has been shown to accumulate in granulomas with proliferative inflammation. 4′-[methyl-^11^C]-thiothymidine (4DST) is incorporated into the DNA of proliferating cells.	Cyclotron	110 min
Radiolabeled choline analogs	^11^C-choline; ^18^F-choline; ^18^F-fluoroethyl-choline	These analogs are taken up in the synthesis of phosphatidylcholine, a cell membrane component.	Cyclotron	20 min
Radiolabeled nitroimidazoles	^18^F-fluoromisonidazole (FMISO)	Localizes to areas with upregulation of hypoxia-inducible factor, which has been shown to be overexpressed in sarcoid granulomas.	Cyclotron	110 min
Radiolabeled amino acid compounds	^11^C-methionine	Increased uptake in areas of enhanced amino acid metabolism, such as areas of inflammation.	Cyclotron	20 min
Radiolabeled CXCR4 receptor ligand	^68^Ga-pentixafor	High affinity for CXCR4 receptor which has increased expression in areas of inflammation.	Generator	68 min

## Data Availability

No new data were created or analyzed in this study. Data sharing is not applicable to this article.

## Abbreviations

CS	cardiac sarcoidosis
PET	positron emission tomography
FDG	fluorodeoxyglucose
JMHW	Japanese Ministry of Health and Welfare
HRS	Heart Rhythm Society
SNMMI	Society of Nuclear Medicine and Molecular Imaging
CT	computed tomography
ASNC	American Society of Nuclear Cardiology
MRI	magnetic resonance imaging
MPI	myocardial perfusion imaging
SPECT	single-photon emission computed tomography
SUV	standard uptake value
UI	uptake index
SSTR	somatostatin receptor
FLT	fluorothymidine
FMISO	fluoromisonidazole
MIP	maximum intensity projection
CXCR4	C-X-C motif chemokine receptor 4
MI	myocardial infarction
